# Inhibitory receptors for HLA class I as immune checkpoints for natural killer cell-mediated antibody-dependent cellular cytotoxicity in cancer immunotherapy

**DOI:** 10.1007/s00262-022-03299-x

**Published:** 2022-10-19

**Authors:** Nicky A. Beelen, Femke A. I. Ehlers, Gerard M. J. Bos, Lotte Wieten

**Affiliations:** 1 Department of Transplantation Immunology, Maastricht University Medical Center+, P. Debeyelaan 25, PO Box 5800, 6202 AZ Maastricht, The Netherlands; 2 GROW, School for Oncology and Developmental Biology, Maastricht University, Maastricht, The Netherlands; 3 Department of Internal Medicine, Division of Hematology, Maastricht University Medical Center+, Maastricht, The Netherlands

**Keywords:** NK cell, HLA, KIR, NKG2A, ADCC, Immunotherapy, Cancer

## Abstract

Natural killer (NK) cells mediate potent anti-tumor responses, which makes them attractive targets for immunotherapy. The anti-tumor response of endogenous- or allogeneic NK cells can be enhanced through clinically available monoclonal antibodies that mediate antibody-dependent cellular cytotoxicity (ADCC). NK cell activation is regulated by interaction of inhibitory receptors with classical- and non-classical human leukocyte antigens (HLA) class I molecules. Inhibitory receptors of the killer immunoglobulin-like receptor (KIR) family interact with HLA-A, -B or –C epitopes, while NKG2A interacts with the non-classical HLA-E molecule. Both types of inhibitory interactions may influence the strength of the ADCC response. In the present review, we provide an overview of the effect of inhibitory KIRs and NKG2A on NK cell-mediated ADCC, which highlights the rationale for combination strategies with ADCC triggering antibodies and interference with the NK cell relevant inhibitory immune checkpoints, such as KIR and NKG2A.

## Natural killer cells are potent mediators of antibody-dependent cellular cytotoxicity

Natural killer (NK) cells are innate immune cells that can mediate potent cytotoxic responses against cancer cells without the need for prior exposure to tumor-associated antigens. NK cells recognize a potential target cell by a broad array of membrane-associated receptors that interact with activating- or inhibitory ligands expressed by the target cell [1]. NK cell effector function is triggered when the net result of engagement via activating NK cell receptors is stronger than the inhibitory signals provided by the target cell [2]. NK cells can distinguish diseased cells from healthy cells through enhanced levels of activating ligands on virally infected or malignantly transformed cells. Healthy cells normally do not express high levels of these molecules as most activating NK cell ligands are molecules associated with cellular stress, malignant transformation or viral infection [1, 2]. The inhibitory receptors, on the other hand, set the threshold for NK cell activation. The most important group of inhibitory ligands are the Human Leukocyte Antigen (HLA) class I molecules expressed on virtually every healthy cell and which protect them from killing by NK cells [1, 2].

NK cells can mediate several effector functions and the most well-known is the killing of diseased cells through the release of granules containing perforin and granzymes or in a death receptor-dependent manner. In addition, they mediate helper functions through the release of cytokines like IFNγ that promote type 1 anti-tumor immunity [2]. NK cells are also potent mediators of antibody-dependent cellular cytotoxicity (ADCC). NK cell ADCC is mediated by the binding of the low affinity Fc-receptor, FcɣRIIIa or CD16, with antibodies bound to target cells [1]. FcɣRIIIa preferably interacts with antibodies of the IgG1 or IgG3 isotype in humans and the glycosylation- and fucosylation status of the antibody have been shown to impact the strength of the response [3]. Genetic variation of the FcɣRIIIa receptor can further influence the response and 234 single nucleotide polymorphisms (SNP) have been described in the *FCGR3A* gene, in coding and in non-coding regions [4]. The FcɣRIIIA-F158V polymorphism (rs396991) is the best characterized SNP in the *FCGR3A* gene with a functional effect. It is the result of a single nucleotide substitution from T to G at cDNA nucleotide position 559 leading to two different FcɣRIIIa allotypes: the high affinity variant with a valine (V) on amino acid position 158 and a lower affinity variant with a phenylalanine (F) at amino acid position 158 [5]. FcɣRIIIa signals via an intracellular CD3Ϛ domain and interaction with the Fc part of an antibody, bound to its target, provides a very strong activating signal to the NK cell. Engagement of only the FcɣRIIIa with an ADCC-inducing antibody is sufficient to trigger NK cell activation, which is in contrast to most other activating NK cell receptors for which signaling via at least two activating receptors is needed [6]. In a clinical trial with the ADCC-inducing antibody isatuximab, FcɣRIIIa 158 V correlated with a prolonged progression-free survival in relapsed or refractory multiple myeloma, which was mechanistically supported in in vitro assays showing enhanced NK cell-mediated ADCC [7]. This property and the option to enhance the NK cell response in a tumor-specific- or tumor-associated manner makes the use of ADCC-inducing antibodies a popular way to therapeutically boost the NK cell anti-tumor response.

## Regulation of NK cell function by interaction of inhibitory NK cell receptors and HLA class I ligands

NK cells express a variety of receptors interacting with HLA class I ligands, among them activating- and inhibitory members of the family of killer immunoglobulin like receptors (KIRs) and NKG2A. Like their HLA ligands, the KIR genes are highly polymorphic. Therefore, many different protein variants and haplotypes exist, and KIR repertoires are further shaped by copynumber variation. Activating family members are named KIRxDSx as they possess a short (S) intracellular ITAM domain providing activating intracellular signaling. Depending on the presence of 2 or 3 extracellular immunoglobulin domains (D) they are named KIR2DSx or KIR3DSx [8]. Activating KIRs can interact with HLA class I, e.g., KIR2DS1 and KIR2DS2 with HLA-C and KIR2DS4 with HLA-C*05:01 [9], but the ligands for several of the other activating KIRs remain elusive. Inhibitory KIR (iKIR) family members express a long (L) intracellular domain leading to an inhibitory signaling cascade upon ligand interaction, which names them KIRxDLx and iKIRs typically recognize HLA class I molecules. The most frequently studied are KIR2DL1 interacting with HLA-C alleles possessing the C2 epitope characterized by a Lysine at aa position 80; KIR2DL2 or KIR2DL3 interacting with HLA-C alleles having the C1 epitope with a Asparagine at aa position 80 [10] and KIR3DL1 binding to HLA-B alleles with a Bw4 epitope or to HLA-A23, -A24 or –A32 [11].

iKIRs have a dual role regulating NK cell activation. The interaction between iKIRs and their HLA class I ligands results in an enhanced potential of the NK cell to respond to diseased cells upon engagement with an activating ligand, a process called “NK cell licensing” [12]. As a result, NK cells that do not express any inhibitory receptor for HLA class I are considered to be hyporesponsive unless they receive a very strong activating signal. Licensed NK cells more vigorously respond to a potential target cell [12, 13]. While iKIRs promote NK cell function through licensing, they also act as strong inhibitory immune checkpoints to control effector function of licensed NK cells. By doing so, iKIRs protect healthy cells from being killed by NK cells through the binding to their respective HLA class I ligands abundantly present on a healthy cell. In contrast to T cells, NK cells recognize HLA epitopes rather than individual HLA alleles, though they can sense to some extend alterations in the HLA peptidome [9]. This is also the reason why NK cells do not cause graft versus host pathology in an allogenic setting with HLA discrepancy which is in sharp contrast to T cells and a great benefit when exploiting NK cells for immunotherapy purposes [14].

The heterodimer CD94:NKG2A interacts with the non-classical HLA class I molecule, HLA-E. With only two frequently occurring protein variants, HLA-E is much less polymorphic than the classical class I molecules HLA-A, -B and –C [15]. While iKIRs are typically expressed on the highly mature NK cell subsets and exclusively on the cytotoxic CD56^dim^ subset, NKG2A can also be found on less mature NK cells as well as on the cytokine producing CD56^bright^ subset. NK cells can (co-)express one or a combination of inhibitory receptors, but, the percentage of NKG2A expressing NK cells in peripheral blood is usually much higher than the percentage of KIR expressing NK cells (20–80% for NKG2A vs. 0–15% for iKIRs) [16]. Like the iKIRs, NKG2A is involved in NK cell licensing and it sets the activation threshold for licensed NK cells and protects HLA-E expressing cells from killing by NK cells [15].

## NKG2A and iKIRs as inhibitory immune checkpoint controllers for NK cell anti-tumor reactivity

In the quest to cure cancer, there is a booming interest in targeting inhibitory immune checkpoint molecules like PD1 and CTLA-4, which even resulted in the awarding of the Nobel Prize for medicine in 2018 to Allison and Honjo. In the past years, many other inhibitory immune checkpoint receptors have been identified, among them TIM-3, LAG-3, TIGIT and CD96. Under homeostatic conditions, these molecules mediate immune tolerance by providing negative feedback loops and controlling excessive immune cell activation. The downside of these receptors is that they also negatively impact anti-tumor immunity as their inhibitory ligands are frequently expressed on tumor cells or tumor-accessory cells, and the receptors themselves are often expressed on tumor infiltrating T cells (TILs), or on T cells in tumor draining lymph nodes and this expression has been associated with an exhausted functional T cell profile [17]. Immunotherapy with monoclonal antibodies blocking the interaction between the receptors and their ligands in the tumor itself or in the draining lymph node has been shown to enhance priming of anti-tumor CD4 and CD8 T cells and to release TILs from inhibition in animal models as well as in human [17]. The enormous potential of targeting these inhibitory receptors has been clearly exemplified in several of the clinical studies performed in humans with for example nivolumab, ipilimumab or pembrolizumab, that may provide clinical responses in some patients [18].

In contrast to the effect on T cells, the role of those prototypic immune checkpoint molecules in NK cells is much less clear and there is a lot of debate on, for example, the functional relevance of PD1 for NK cells [19]. Regarding its ligand PD-L1, one study indicates that PD-L1 targeting may be beneficial in PD-L1-negative tumors and that this is mediated through the direct binding of monoclonal antibodies to tumor-induced PD-L1 expression on the NK cells [20]. Besides PD-L1, NKG2A and the iKIRs are additional controllers of NK cell reactivity against self- and non-diseased cells and can therefore also be considered as critical inhibitory immune checkpoint receptors for NK cells. Hence, potentiating NK cell anti-tumor responses via the blockade of inhibitory interactions between KIRs or NKG2A and their HLA ligands is a highly interesting approach to boost the curative potential of NK-based therapies. Antibodies like lirilumab (anti-KIR) and monalizumab (anti-NKG2A) have been developed and tested for this purpose [21, 22]. While many tumors reduce or even completely lose HLA class I expression to escape from CD8 T cells, they frequently maintain or even enhance expression of HLA-E, presumably to protect them from killing by NKG2A-positive NK cells (thus the majority of NK cells) [23]. This makes NKG2A an especially interesting candidate to further target in clinical trials [22]. Currently, there are 16 trials registered at Clinicaltrials.gov that study the potential for monalizumab in cancer. In addition, lirilumab is being used in 13 clinical trials. Both monalizumab and lirilumab are utilized in combination with other therapeutic agents in the majority of these studies. Although these types of combination therapies have been well tolerated by patients, they do not yet result in optimal stimulation of NK cell anti-tumor efficacy and the exact combination resulting in optimal NK cell anti-tumor efficacy remains to be determined. While the stop of the INTERLINK-1 Phase 3 trial studying monalizumab with cetuximab (NCT04590963) was recently announced, other studies with different combination therapies, such as monalizumab with durvalumab, are ongoing (e.g., NCT05221840 and NCT02671435). It will be interesting to unravel the underlying reasons for the lack of improved survival and to understand how the treatment affected NK cells.

When NK cells are used in an allogeneic setting, interaction between iKIR and HLA can also be reduced through selection of so-called KIR-ligand mismatched donors [24]. The beneficial effect of a KIR-ligand mismatch became clear from animal- and human studies showing an NK mediated improved clinical outcome in leukemia patients upon haploidentical stem cell transplantation when KIR-ligand mismatched donors were selected [25]. Our group obtained comparable results in a mouse breast cancer model, providing evidence that the concept may also be relevant for solid tumors [26]. Furthermore, we and others demonstrated in in vitro models that KIR-ligand mismatched NK cells were more responsive to HLA class I expressing tumor cells, even in a setting where the NK cells were highly activated by IL-2 [27–29].

## Triggering ADCC as a way to potentiate the NK cell anti-tumor response

The development of the hybridoma technology as well as more recent approaches to produce and optimize monoclonal antibodies for therapeutic purposes has revolutionized the cancer immunotherapy field. A plethora of clinical grade antibodies are currently available and many of them act in a multimodal way; for example, via deprivation of tumor growth factors, by triggering complement-dependent cytotoxicity (CDC), by promoting opsonization and subsequent degradation by macrophages, by potentiating the immune response through blockade of inhibitory receptors, and/or by triggering Fc receptor mediated ADCC [30]. Since monoclonal antibodies can be used in an off the shelf manner, they are attractive candidates to combine with other immunotherapeutic strategies that exploit the anti-tumor function of endogenous NK cells or allogeneic NK cells in a transplantation or adoptive transfer setting. Several monoclonal antibodies have been shown to induce ADCC mediated by NK cells and frequently studied examples are trastuzumab for Her2 expressing tumors, cetuximab (anti-EGFR), rituximab or obinutuzumab (anti-CD20) for B cell malignancies, daratumumab (anti-CD38) or elotuzumab (anti-CS1) in multiple myeloma (reviewed in [31]).

## Influence of KIR and NKG2A on NK cell-mediated antibody-dependent cellular cytotoxicity

**Fig. 1:**
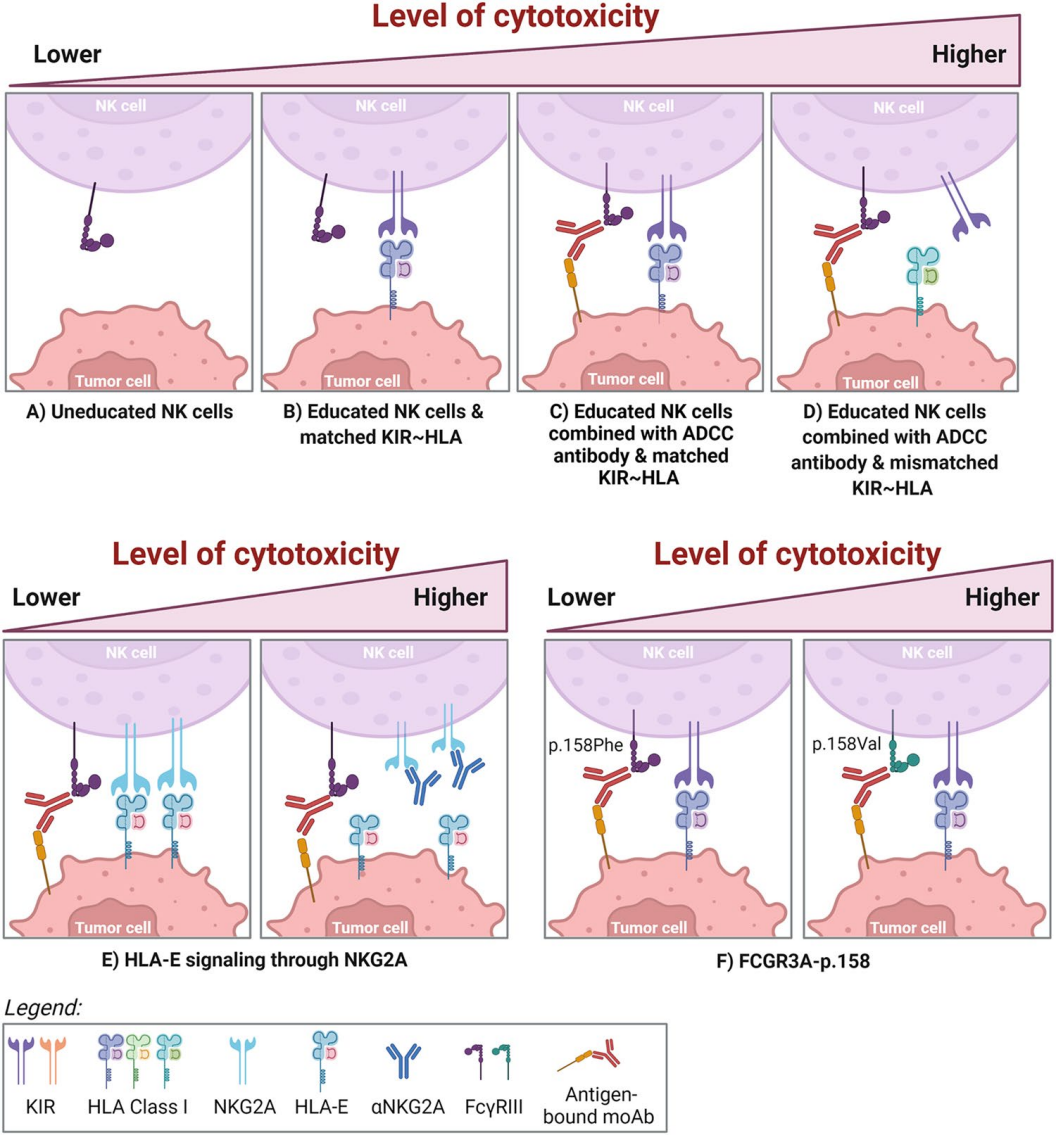
The effect of NK cell education status, KIR/HLA interaction, NKG2A/HLA-E interaction, and *FCGR3A*-p.158 genotypes on the efficacy of ADCC. Level of NK cell-mediated anti-tumor cytotoxicity increases from left to right. **A** Uneducated NK cells require stimulation by ADCC-inducing antibodies in order to target tumor cells. Moreover, maximal ADCC responses require NK cell education. **B**–**C** In educated NK cells, KIR-ligand matching provides a strong inhibitory signal, which can be overcome by adding ADCC-inducing antibodies. However, different levels of inhibition have been described for different KIR/HLA interactions. **D** Mismatched educated NK cells combined with ADCC-inducing antibodies have the highest level of cytotoxicity. **E** High levels of HLA-E can lower cytotoxic potential even though ADCC-inducing antibodies are present. Anti-NKG2A antibodies, such as monalizumab, could effectively block this interaction. **F** Additionally, the high affinity variant *FCGR3A*-p.158Val induces stronger ADCC, independent of educational status. Created with BioRender.com

In the setting where tumor-specific ADCC triggering antibodies are used to promote NK cell activation, NK cells will receive a very strong activating signal which may override the inhibitory effects of KIR/NKG2A interaction with HLA class I. Several in vitro studies have explored the influence of KIR-ligand interaction and licensing status of the NK cell on the outcome of ADCC (Table 1 and Fig. 1). Most studies used rituximab (anti-CD20) to potentiate the NK cell response against B cell leukemias, in neuroblastoma ADCC can be triggered with anti-GD2, while in multiple myeloma daratumumab (anti-CD38) was used. All studies that addressed the role of licensing status of the NK cell demonstrated that both licensed- and non-licensed NK cells could mediate ADCC upon engagement of an antibody coated target cell [32–34]. Importantly, this showed that the hyporesponsive NK cell subsets, lacking expression of iKIRs, are also effective effector cells and can contribute to the overall anti-tumor response of bulk NK cells when an ADCC triggering antibody is present. Lisovsky et al.[32] separately examined the contribution of education to antibody-dependent NK cell activation and found that education through KIR3DL1 and KIR2DL1, but not KIR2DL3, increased antibody-mediated secretion of IFN-γ and CCL4, compared to uneducated counterparts.

**Table 1:** Impact of inhibitory killer immunoglobulin-like receptors on NK cell-mediated ADCC

References	Study summary	Results summary	Effect through
[42]	In vitro study with CD20 positive B-lymphoblast cell lines and triggering of NK cell ADCC by rituximab	HLA, but not CD20 expression on the target cell correlated with rituximab-induced ADCC. Anti-KIR antibodies enhanced ADCC	KIR-ligand interaction
[43]	In vitro study testing effect of NK mediated ADCC by anti-CD19 (XmAb5574) or anti-CD33 (lintuzumab) against NK resistant leukemia cell lines or primary leukemia cells	Antibodies enhanced the killing of the cell lines. Blockade of KIR-ligand interaction with a pan-HLA class I moAb further enhanced the response	KIR-ligand interaction
[44]	408 follicular lymphoma patients receiving 4-weekly rituximab as induction therapy. Clinically responding patients (n = 289) were randomized to receive 13-week rituximab maintenance therapy vs non-maintenance until progression	No overall-benefit for rituximab maintenance. Genotypic presence of KIR2DL2 and C1 as well as KIR3DL1 and Bw4 associated with improved outcome and duration of the response after maintenance therapy with rituximab	KIR-ligand interaction
[36]	245 neuroblastoma patients receiving 3F8 moAb (anti-GD2). Comparison of predicted weak vs strong KIR3DL1-Bw4 interactions	Lack of KIR3DL1 expression or absence of KIR-Bw4 ligands improved overall- and progression-free survival. Patients with predicted weak KIR3DL1-Bw4 interactions had superior outcomes than patients with strong interactions	KIR-ligand interaction
[37]	In vitro study exploring the effect of KIR on ADCC induced by rituximab or obinutuzumab (anti-CD20, glycoengineered for higher affinity binding to FcɣRIIIa	NK cells from individuals having all three iKIRs poorly killed rituximab bound KIR-ligand matched B cells. NK cells from individuals lacking one or more iKIRs more potently killed KIR-ligand incompatible B cells in presence of rituximab. NK cells with KIRs lacking the ligand on the target cell were most potent. ADCC by obinutuzumab was not negatively influenced by KIR-ligand interaction	KIR-ligand interaction
[35]	In vitro analysis of rituximab-induced ADCC of healthy donor NK cells with known KIR genotyping against a panel of EBV B cell lymphoblastoid target cells with various HLA class I alleles	Stronger inhibitory modulator effects on rituximab-dependent NK cell degranulation through KIR2DL1/HLA-C C2 + and KIR3DL1/HLA-B Bw4 + signaling, compared to KIR2DL2/3 with HLA-C C1 + . Inhibitory signaling through KIR2DL1 and KIR3DL1 brought degranulation levels down to the baseline of uneducated counterpart	KIR-ligand interaction
[41]	In vitro analysis of daratumumab-induced ADCC against myeloma cell lines and primary cells in combination with blockade of KIR-ligand interaction with anti-KIR IPH2102 (lirilumab)	KIR blockade by IPH2102 enhanced daratumumab-induced ADCC. This was especially evident in patients carrying the low affinity (158F) FcɣRIIIa receptor variant	KIR-ligand interaction
[45]	In vitro study with breast cancer cell lines (SKBR3, T47D, MCF-7) and trastuzumab	Trastuzumab mediates ADCC against HER2 overexpressing cell lines which is irrespective of the NK cell donor genotype	KIR-ligand interaction
[7]	Phase IIb clinical trial including 57 patients with (RR)MM receiving Isatuximab-Lenalidomide-Dexamethasone combination, supported by in vitro cytotoxicity assays	Most prolonged PFS with patients carrying KIR3DL2 + HLA-A3/11 + and FCGR3A 158 V polymorphism combination (Not due to education), which was recapitulated in in vitro ADCC assays. Suppressed NK cell ADCC with KIR2DL1 + HLA-C2 + interaction	KIR-ligand interaction
[46]	In vitro study using NK cells expanded on K562-mbIL15-41BBL cells. ADCC induction via rituximab against 721.221 target cells	Rituximab-induced ADCC and enhanced killing of target cells. KIR-ligand mediated inhibition partially persisted	KIR-ligand interaction
[38]	In vitro and xenograft in vivo models with breast cancer cell line SKBR3 and trastuzumab	KIR2DL4 synergizes with FcɣRIIIa to enhance NK cell degranulation. HLA-G/KIR2DL4 interaction impairs trastuzumab induced ADCC in HER2 positive breast cancer	KIR-ligand interaction
[33]	In vitro analysis of ADCC triggered by anti-GD2 of IL-2 activated NK cells against neuroblastoma cells	Degranulation of licensed NK cells and KIR-ligand mismatched NK cells was higher than of non-licensed or matched counterparts	KIR-ligand interaction Educational status
[34]	In vitro study testing the effect of anti-GD2 and KIR-ligand mismatching on ADCC against neuroblastoma cells	Both licensed and unlicensed NK cells can mediate ADCC. KIR ligand interaction inhibited ADCC of licensed NK cells	KIR-ligand interaction Educational status
[28]	In vitro study exploring daratumumab-induced ADCC against myeloma cell lines and the influence of the tumor microenvironment on the response	Daratumumab mediates ADCC of licensed and non-licensed NK cells, also under conditions mimicking the suppressive tumor microenvironment. NKG2A does not strongly influence the response. KIR-ligand mismatched NK cells mediate the most potent anti-myeloma response	KIR-ligand interaction Educational status NKG2A education
[32]	In vitro analysis of antibody *(anti-HIV envelope Env and Env expressing target cells*) induced ADCC and antibody-dependent NK cell activation using PBMCs from HIV-1 uninfected donors	NK cell education does not influence ADCC levels. Education through KIR3DL1 and KIR2DL1, but not 2DL3, increased antibody-dependent NK cell activation (ADNKA), when compared to counterparts that were not able to educate through these KIRs	Educational status

All studies that addressed the potential inhibitory effect of KIR-ligand interaction on ADCC concluded that NK cells with KIRs that do not encounter their HLA ligands on the target cell mediate more potent responses than their matched counterparts [7, 28, 33, 35–37]. Kohrt et al. [37] observed that inhibition via KIR-ligand interaction was relevant when rituximab was used to trigger ADCC against CD20 expressing B cells but not when the Fc optimized variant obinutuzumab was used to trigger ADCC, illustrating that the exact nature of the antibody is important as well [37]. Of relevance, there are indications that not all KIR-ligand interactions mediate similar levels of ADCC inhibition. Makanga et al. [35] described that KIR2DL1/HLA-C C2 + and KIR3DL1/HLA-B Bw4 + signaling strongly mediated NK cell hypo-responsiveness to rituximab, whereas the effects through KIR2DL2/3 with HLA-C C1 + were less pronounced. This is consistent with findings by Sun et al. [7], where isatuximab-mediated ADCC was suppressed by KIR2DL1/HLA-C2 + interaction.

Non-classical HLA class I may also affect efficacy of ADCC. HLA-G interaction with KIR2DL4, which is present in all KIR haplotypes, inactivates NK cells and desensitizes breast cancer cells to trastuzumab treatment. In HLA-G-negative tumors, KIR2DL4 promotes ADCC through an IFN-γ-mediated feedback circuit until IFN-γ eventually upregulates PD-L1 on cancer cells [38]. Therefore, interfering with HLA-G/KIR2DL4 signaling combined with PD-L1/PD-1 targeting may potentiate trastuzumab treatment in those resistant cancers.

Albeit its frequent expression on NK cells, the role of NKG2A is relatively unexplored. Our group demonstrated that NK cells licensed via NKG2A mediated more potent anti-myeloma responses than unlicensed NK cells [28]. Furthermore, co-expression of NKG2A did not reduce the magnitude of the response against target cells expressing low- or intermediate levels of HLA-E when the NK cells were highly activated by IL-2 and in the presence of an ADCC triggering monoclonal antibody (moAb). High levels of HLA-E expression on target cells did however significantly reduce the NK cell response, indicating that the density of the inhibitory ligands is important as well [28].

NK cells, like many other immune effector cells, can be severely hampered in mediating their anti-tumor effects by an immunosuppressive tumor microenvironment (TME) including factors like hypoxia, prostaglandin E2, lactate and suppressive tumor-accessory cells [39, 40]. This emphasizes the need to develop combination strategies to facilitate anti-tumor immune effector functions. In vitro data, as well as several clinical studies encouragingly show that ADCC mediating moAb can promote NK function even under suppressive conditions (reviewed in [29]). Given the strong inhibitory effects of especially KIR-ligand interaction, interfering with these immune checkpoint molecules may be a way to further enhance the response. In a setting where endogenous NK cells are targeted, this can be done by using moAb with specificity for KIRs (e.g., lirilumab), as has been done in for example myeloma [41] or in tumors with high levels of HLA-E by using monalizumab [22]. In an allogeneic setting, selection of KIR-ligand mismatched donors may be an alternative strategy. Though, the additive effect of KIR-ligand mismatching in a transplant setting is still controversial and seems to be highly dependent on the exact transplantation setting. Furthermore, it may be interesting to further explore the synergistic effect of targeting KIR/NKG2A when chimeric antigen receptors (CARs) are used to redirect and enhance the NK cell anti-tumor response an alternative for ADCC triggering antibodies.

In either case, minimizing NK cell inhibition by interfering with KIR/NKG2A or any other relevant inhibitory immune checkpoint receptors, in combination with an approach to maximize NK cell activation via engagement of CD16 or a CAR may help to potentiate the NK cell anti-tumor effects which will be especially relevant for tumors with a highly suppressive TME.
